# Causal relationships between gut microbiota and IgA nephropathy: evidence from Mendelian randomization and microbiome validation

**DOI:** 10.1080/0886022X.2025.2522979

**Published:** 2025-06-25

**Authors:** Xin Wang, Jiong Liu, Wuda Huoshen, Jing Liu, Xue Qiao, Hong Zhang, Xu-Jie Zhou

**Affiliations:** a Renal Division, Peking University First Hospital, Beijing, China; b Kidney Genetics Center, Peking University Institute of Nephrology, Beijing, China; c Key Laboratory of Renal Disease, Ministry of Health of China, Beijing, China; d Ministry of Education, Key Laboratory of Chronic Kidney Disease Prevention and Treatment (Peking University), Beijing, China; e State Key Laboratory of Vascular Homeostasis and Remodeling, Peking University, Beijing, China; f School of Stomatology, Southwest Medical University, Luzhou, China; g New Cornerstone Science Laboratory, CAS Key Laboratory of Biomedical Effects of Nanomaterials and Nanosafety and CAS Center for Excellence in Nanoscience, National Center for Nanoscience and Technology of China, Beijing, China; h State Key Laboratory of Natural and Biomimetic Drugs, School of Pharmaceutical Sciences, Peking University, Beijing, China

**Keywords:** IgA nephropathy, Mendelian randomization, genome-wide association study, gut-kidney axis, microbiota-disease causality

## Abstract

**Background:**

Emerging evidence links gut microbiota strongly with IgA Nephropathy (IgAN). However, the causal role of specific gut microbiota in IgAN remains unclear. This study used a two-sample Mendelian randomization (MR) approach, validated with 16S rRNA datasets, to identify these causal relationships.

**Methods:**

We performed MR analysis using genetic instruments for 412 gut microbiota taxa from genome-wide association studies (GWAS) as exposures and IgAN GWAS data as outcomes. The inverse-variance weighted method was used as the primary analysis, supplemented by MR-Egger regression, weighted median methods, and Cochran’s Q test to assess pleiotropy and heterogeneity. Significant findings were validated using reverse, multivariable, and mediation MR analyses. Results were validated using genus-level 16S rRNA datasets with batch correction (ConQuR), and microbial function was inferred *via* PICRUSt2.

**Results:**

Three gut microbiota species were protective against IgAN: *s_Alistipes_senegalensis* (OR = 0.64, *p* = .002), *s_Ruminococcus_bromii* (OR = 0.75, *p* = .040), and *s_Bilophila_unclassified* (OR = 0.68, *p* = .040). Six species were associated with increased IgAN risk, including *g_Barnesiella* (OR = 1.32, *p* = .030) and *s_Rothia_mucilaginosa* (OR = 1.52, *p* = .040). After multiple-testing correction, significant associations persisted for *s_Alistipes_senegalensis* (*p* = .043), *s_Bacteroides_clarus* (*p* = .035), and *s_Bilophila_unclassified* (*p* = .002). Sensitivity analyses confirmed robust results without pleiotropy or heterogeneity. Genus-level validation confirmed consistent microbial shifts. Functional predictions showed upregulation of carbohydrate/fatty acid metabolism and downregulation of the urea cycle.

**Conclusions:**

This study reveals specific gut microbes and metabolic pathways potentially driving IgAN, offering novel biomarkers and therapeutic targets for microbiome-based interventions.

## 1. Introduction

IgA nephropathy (IgAN) is one of the most prevalent primary glomerulonephritides worldwide, characterized by the deposition of IgA-containing immune complexes in the glomerular mesangium [1]. The disease trajectory varies widely across patients, ranging from asymptomatic hematuria to progressive kidney failure [2]. Despite extensive research on the immunologic and genetic underpinnings of IgAN, a comprehensive understanding of all contributing factors remains elusive [3].

In parallel, the human gut microbiota (GM) has emerged as a key regulator in many diseases [4]. Recent studies have implicated the microbiota in modulating the immune system [5], metabolism [6], and even response to drug therapies [7]. Among kidney diseases in particular, there is a growing body of work exploring the microbiome-renal axis, highlighting how changes in the gut microbiome might affect systemic inflammation and immune regulation [8,9]. Genome-wide association studies (GWAS) have contributed greatly to unraveling potential genetic determinants of microbial composition, identifying loci that may influence both host genetics and microbial abundance [10]. Mendelian randomization (MR) has become an increasingly popular tool to investigate causal relationships in this domain, as it uses genetic variants as instrumental variables (IVs) to infer causality [11]. This approach is particularly valuable for alleviating the confounding and reverse causation issues that often plague observational studies [12].

To date, numerous GWAS have examined microbiota-associated genetic variants, with some focusing on the potential roles of specific bacterial taxa in the context of immune-mediated disorders [13]. However, few have attempted a bidirectional approach to ascertain whether the relationship between specific taxa and IgAN is causal from the perspective of both exposure → outcome and outcome → exposure. Moreover, the mechanistic pathways through which any identified bacteria might influence IgAN remain underexplored. For instance, serum IgA plays a central role in IgAN pathogenesis [14], yet its mediation effect in the gut microbiota–IgAN relationship is sometimes assumed without rigorous testing.

This study, therefore, aims to clarify the potential causal links between specific gut microbial taxa and IgAN using a bidirectional two-sample MR framework. By leveraging large-scale GWAS summary statistics of gut microbiota and IgAN, we seek to determine whether certain microbes genetically predispose individuals to IgAN or whether IgAN status influences gut microbiota composition in return. In addition, we investigate whether serum IgA levels might serve as a mediating factor in any observed relationships, using a two-step MR mediation analysis. Finally, we attempt to validate our findings by integrating publicly available gut microbiota sequencing datasets from different cohorts, taking measures to mitigate batch effects and ensure reproducible and reliable conclusions. We provide a refined methodology for MR analysis, emphasizing reproducibility, and present our results in a manner that is both statistically rigorous and accessible to readers. By doing so, we aim to add to the growing literature on the gut microbiota’s role in IgAN pathophysiology and highlight potential biomarkers or therapeutic targets for future interventions.

## 2. Materials and methods

### 2.1. GWAS database

We obtained gut microbiota data (GM) from a previous study conducted by Esteban et al. [15], where 412 microbiotas in the gut were assessed in 7738 participants. The authors performed a GWAS that included 207 taxa, spanning multiple taxonomic levels (5 phyla, 10 classes, 13 orders, 26 families, 48 genera, and 105 species), along with 205 pathways reflecting microbial composition and activity. From this dataset, we selected genetic instruments based on three criteria: (a) genome-wide significance (*p* < 1 × 10^−5^), (b) independence (linkage disequilibrium clumping with r^2^ < 0.001 and kb < 10,000), and (c) exclusion of the HLA region (chromosome 6, 25–35 Mb) [16].

Summary statistics for IgAN were drawn from a large-scale GWAS encompassing 11 European cohorts (5556 cases and 21,178 controls), where the diagnosis of IgAN was confirmed by dominant mesangial IgA staining in kidney biopsy tissues [17]. Patients with secondary causes were excluded. The genetically instrumented variants for IgAN were also filtered to ensure independence and significance, though with thresholds relevant to IgAN and consistently excluding the HLA region.

**Figure 1.**
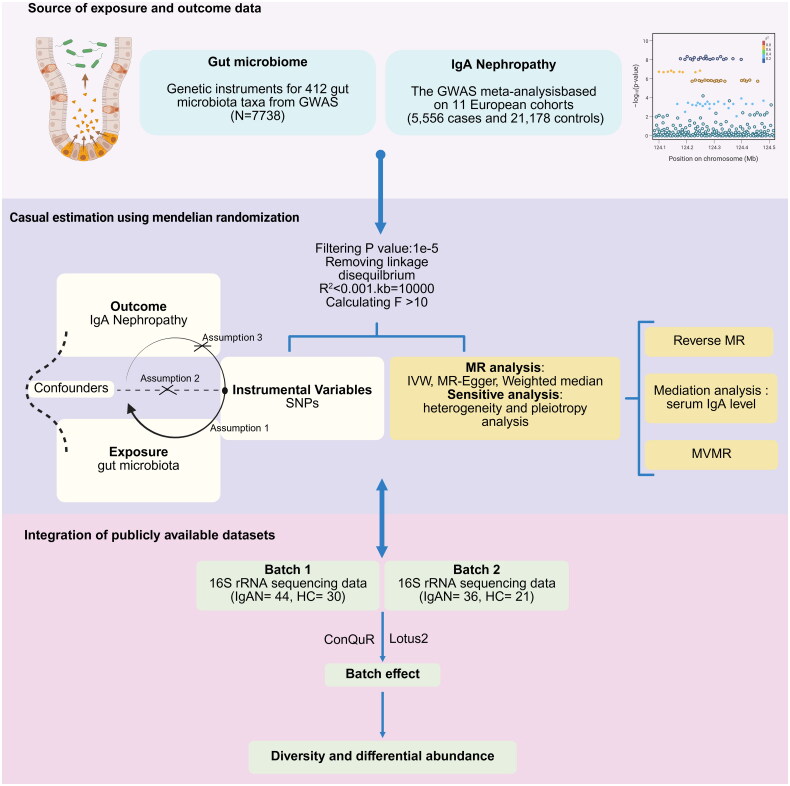
Schematic workflow of the study design investigating the relationship between gut microbiome and IgA nephropathy. The study integrates data from two main sources: gut microbiome genetic instruments from GWAS (*N* = 7738) analyzing 412 gut microbiota taxa, and IgAN GWAS meta-analysis data from 11 European cohorts (5556 cases and 21,178 controls). MR analysis was performed after filtering (*p* < 1e − 5, R^2^ < 0.01, kb = 10,000, *F* > 10) to assess causality. Multiple MR methods including IVW, MR-egger, and weighted median were employed, along with sensitivity and pleiotropy analyses. The study also incorporated publicly available 16S rRNA sequencing data from two independent batches (batch 1: IgAN = 44, HC = 30; batch 2: IgAN = 36, HC = 21), which were analyzed using ConQuR and Lotus2 for batch effect correction and subsequent diversity and differential abundance analyses. Mediation analysis was conducted to evaluate the role of serum IgA levels in the causal pathway. Assumption 1: the genetic IVs are strongly associated with exposure. Assumption 2: the genetic IVs are not associated with confounders linked to the selected exposure and outcome. Assumption 3: the genetic IVs influence the outcome only through the exposure. Abbreviations: ConQuR: conditional quantile regression; IgAN: IgA nephropathy; MR: Mendelian randomization; IVW: inverse variance weighted; HC: healthy control; GWAS: genome-wide association studies.

We exclusively used European data due to the availability of high-quality GWAS datasets for both gut microbiota and IgAN, ensuring robust statistical power and minimizing population stratification bias. An overview of the study design is presented in Figure 1.

### 2.2. Statistical analysis and sensitivity analysis

We conducted a two-sample MR analysis using the above GWAS summary statistics for gut microbiota and IgAN to estimate potential causal effects in both directions: (1) GM as exposure and IgAN as outcome and (2) IgAN as exposure and GM as outcome. This bidirectional design helps clarify the temporal relationship between exposure and outcome, thereby limiting the risk of reverse causation.

To identify causal relationships, we used the inverse variance weighted (IVW) method as the primary estimator. The IVW method combines Wald ratios of individual single-nucleotide polymorphisms (SNPs) while accounting for their variances, offering a powerful approach under the assumption that all genetic instruments are valid.

Recognizing that no single MR method is free from biases, we complemented the IVW estimates with a suite of additional methods: weighted median, MR-Egger, simple mode, weighted mode, and the Wald ratio. A statistically significant association was indicated by *p* < .05. Importantly, we also evaluated horizontal pleiotropy (i.e. SNPs influencing the outcome *via* pathways other than the exposure of interest) by performing MR-Egger intercept tests. Heterogeneity across SNPs was quantified using Cochran’s Q test, which checks the consistency among individual SNP ratios.

### 2.3. Mediation analysis

To investigate whether any associations between gut microbes and IgAN might be mediated by serum IgA levels, we employed a two-step MR (mediation) analysis. The following three conditions were necessary: (i) A causal relationship between the gut microbiota exposure and the mediator (serum IgA level). (ii) A causal relationship between the mediator (serum IgA) and the outcome (IgAN). (iii) A persistent direct effect between the gut microbiota exposure and IgAN in the absence of reverse causality.

For the IgA-level data, we relied on a GWAS meta-analysis of 22,229 individuals from 16 cohorts, selecting instrumental variables for IgA at *p* < 5 × 10^−8^ with r^2^ < 0.001 [18]. If the above three conditions were not met, we concluded that serum IgA level did not mediate the relationship between a specific gut microbe and IgAN.

### 2.4. Integration of publicly available datasets

To confirm the results from our genetic analyses in actual microbial sequencing profiles, we acquired two independent publicly available datasets. One 16S rRNA dataset was from Dong et al. [19] (Batch 1: IgAN = 44, HC = 30), and another from our own previous report [20] (Batch 2: IgAN = 36, HC = 21). These datasets met our inclusion criteria: they specifically assessed gut microbiota in IgAN and stemmed from distinct geographic regions or used different sequencing platforms, ensuring broader applicability.

To maintain consistency, the datasets were processed uniformly using Lotus2 software [21] following a straightforward pipeline:

Pre-Processing: Removal of low-quality reads and sequencing contaminants.

Sequence Clustering: Grouping reads into OTUs/ASVs based on sequence similarity thresholds.

Taxonomic Assignment: Matching OTU/ASV sequences to reference taxonomies to identify bacterial taxa.

Phylogenetic Tree Construction: Generating a tree for downstream diversity analyses.

Host Genome Removal: Eliminating reads aligning with the human genome.

After processing, data from the two studies were merged. We addressed batch effects using the Conditional Quantile Regression (ConQuR) approach [22], which leverages non-parametric modeling of microbial read counts to preserve clinically relevant signals. By visualizing principal coordinate analysis (PCoA) before and after batch correction, we ensured that differences attributable to batch sources were minimized (no residual significance), allowing reliable comparisons between IgAN patients and controls. PICRUSt2 was utilized to predict metabolic pathways of microbial communities [23].

### 2.5. Statistical analysis

All statistical analyses were performed in R (version 4.3.2). For MR and sensitivity analyses, we used the TwoSampleMR (version 0.5.6) package. The reporting of our MR analysis follows the STROBE-MR guidelines [24]. A completed STROBE-MR checklist is provided in Supplementary Table S5. Group comparisons between IgAN and controls employed the two-sample t-test or Wilcoxon rank sum test for continuous data, and chi-square or Fisher’s exact tests for categorical data. In differential abundance analysis, the ‘vegan’, ‘psych’, ‘microViz’, ‘tibble’, ‘dplyr’, ‘phyloseq’, ‘ggplot2’, ‘ggpubr’, ‘tidyverse’, and ‘deseq2’ packages facilitated alpha and beta diversity calculations, visualization, and statistical comparisons. PERMANOVA (Permutational Multivariate Analysis of Variance) analysis based on Bray-Curtis dissimilarity was performed to assess differences in beta diversity. Genera with differential abundance (*p* < .05 and |log2FC| > log2(1.5); DESeq2) are shown. When multiple tests were performed, we controlled for false positives using the False Discovery Rate (FDR) method. A *p* value < .05 was considered statistically significant, unless otherwise noted.

To ensure reliability and reproducibility:

Validation of Instrument Strength: We computed the F-statistic for each SNP to confirm robust instrument strength.

Removal of Outliers: Any problematic SNPs flagged by outlier tests in robust MR approaches (eg, MR-PRESSO) were examined and removed if deemed invalid.

Detailed Sensitivity Pipeline: In addition to MR-Egger intercept tests, we used leave-one-out analyses to check if any single SNP disproportionately influenced the overall result.

Replication: The integrated analysis with external 16S rRNA/metagenomic datasets provided a layer of real-world validation, facilitating cross-verification of the genetically inferred relationships.

## 3. Results

### 3.1. Genetic causality between gut microbiota and IgA nephropathy

**Figure 2.**
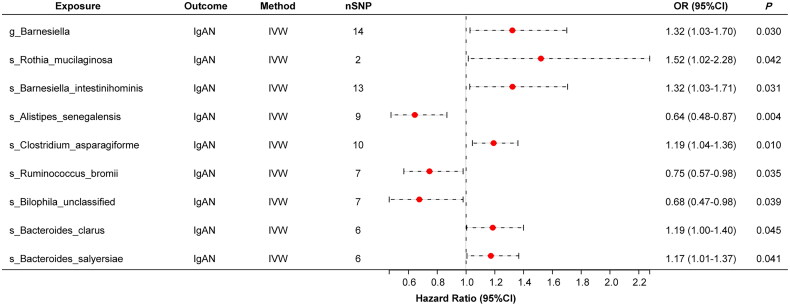
Forest plot of the significant result of Mendelian randomization analysis for gut microbiota and IgA nephropathy risk. The significant result of the causal relationships of gut microbiota on the risk of IgAN. nSNP: total number of instrumental variables used for analysis, or > 1 exposure increases risk of outcome. OR < 1 exposure reduces the risk of the outcome. *p: p* value of the inverse variance weighted method. Abbreviations: SNP: single-nucleotide polymorphism; OR: odds ratios; CI: confidence interval.

We performed a two-sample MR analysis focusing on gut microbes as exposures and IgAN as the outcome. Based on the primary IVW method, nine bacterial taxa exhibited nominally significant associations with IgAN. Among them, *g_Barnesiella*, *s_Rothia mucilaginosa*, *s_Barnesiella intestinihominis*, *s_Clostridium asparagiforme*, *s_Bacteroides clarus*, and *s_Bacteroides salyersiae* were positively associated with IgAN (OR > 1), suggesting increased risk. In contrast, *s_Alistipes senegalensis*, *s_Ruminococcus bromii*, and unclassified *s_Bilophila* showed protective associations (OR < 1) (Figure 2). To solidify these findings, we conducted multiple sensitivity analyses. Neither the MR-Egger intercept test nor Cochran’s Q test revealed substantial horizontal pleiotropy or heterogeneity (*p* > .05 across models) (Figure 2, Figure S1, Tables S1 and S2). However, after applying FDR correction, none of the associations remained statistically significant after correction (q-value > 0.05) (Table S2).

We also performed reverse MR analyses to investigate whether IgAN could causally affect the abundance of these nine taxa. The results did not support a reverse causation scenario for any of the identified microbes (*p* > .05 in all reverse MR tests) (Table S3), reinforcing the notion that specific gut microbial components may play a potentially causal role in elevating or diminishing IgAN risk, rather than vice versa.

### 3.2. Multivariable Mendelian randomization of significant gut microbiota and IgA nephropathy

**Figure 3.**
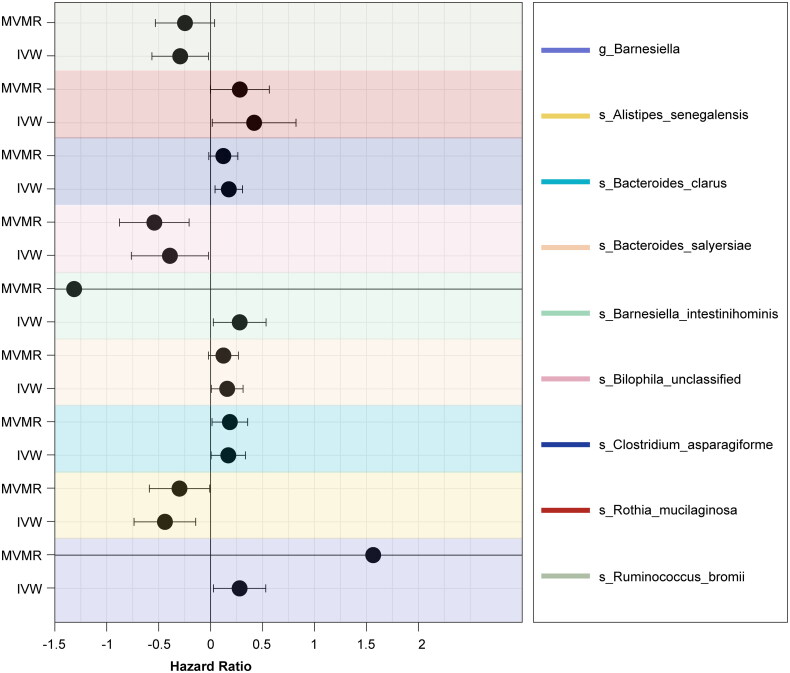
Multivariable Mendelian randomization results of the significant gut microbiota and IgA nephropathy. Abbreviations: MVMR: multivariable Mendelian randomization; IVW: inverse variance weighted.

Recognizing that the gut microbiota is a complex ecosystem, we performed a multivariable MR (MVMR) analysis to account for the interplay among the nine significant taxa within a single model. After adjusting for co-exposures, *s_Alistipes_senegalensis* (IVW: *p* = .043, OR = 0.74, 95% CI = 0.57–0.97), *s_Bacteroides_clarus* (IVW: *p* = .035, OR = 1.20, 95% CI = 1.03–1.40), and *s_Bilophila_unclassified* (IVW: *p* = .002, OR = 0.58, 95% CI = 0.43–0.79) remained significantly associated with IgAN. The sustained significance of these taxa after mutual adjustment suggests they are particularly robust candidates for further exploration (Figure 3). Therefore, even when considering the interactive nature of gut microbial communities, these three species exhibited independent genetic associations with IgAN risk.

### 3.3. Mediation analysis through serum IgA levels

Because IgA dysregulation is central to IgAN, we investigated whether the identified microbial exposures might influence IgAN indirectly *via* serum IgA levels. In the two-step mediation testing, however, none of the significant microbes showed a strong causal link with serum IgA levels (*p* > .05 for all). Moreover, no microbe–IgAN associations could be explained by changes in IgA levels. These findings imply that while IgA is pathophysiologically integral to IgAN, the microbes highlighted here appear to exert their influence *via* pathways independent of directly modulating systemic IgA (Table S4).

### 3.4. Integration of public datasets and taxonomic differences

**Figure 4.**
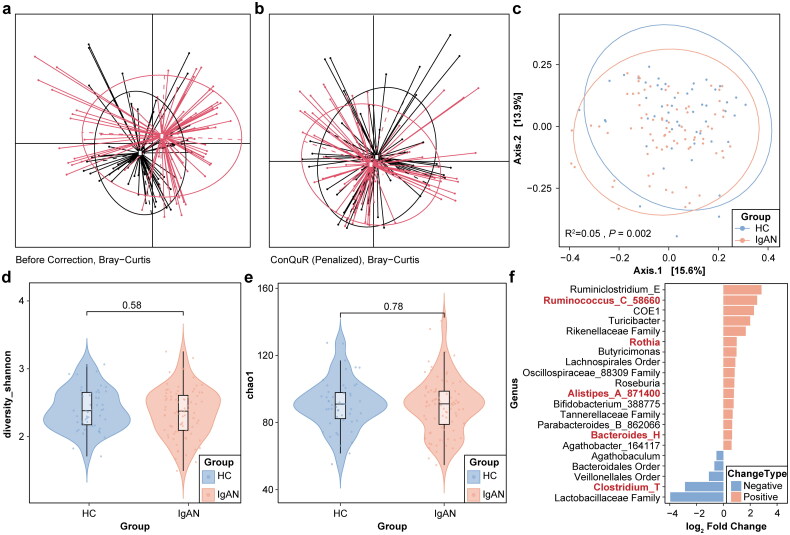
Integrative analysis of gut microbiota data from different batches between IgA nephropathy and healthy controls. For (a,b) PCoA plots clustered by two batch based on raw count data by Bray-Curtis dissimilarity. Each point represents a sample, and each ellipse represents a batch with the centroid indicating the mean. (c) PERMANOVA analysis based on Bray-Curtis dissimilarity was performed to assess differences in beta diversity between groups. (d,e) Alpha diversity was estimated using both the Chao1 index and the Shannon index. Two-sided Wilcoxon rank-sum test was used to assess the statistical significance. (f) Genera with differential abundance (*p* < .05 and |log2FC| > log2(1.5); DESeq2) are shown.

To validate the MR findings, we turned to external cohorts from Dong et al. and our own previous report. Following data processing with Lotus2 and subsequent batch-effect removal by ConQuR, PCoA plots confirmed that combining the two datasets introduced no residual batch-specific clustering (*p* = 1 after correction) (Figure 4 (a,b)). We then analyzed alpha diversity (Chao1 and Shannon indices), comparing IgAN patients and controls. No major differences were noted in either index (Figure 4(d,e)). Using PERMANOVA analysis, however, we observed significant compositional divergence between the IgAN and healthy samples (*p* = .002), suggesting that while overall diversity may be similar, the taxonomic composition differs measurably (Figure 4(c)).

Further differential abundance analysis (*p* < .05, and |log2FC| > log2(1.5); DESeq2) identified multiple genera enriched in IgAN patients, including *Bacteroides_H, Parabacteroides_B_862066, Agathobacter_164117, Roseburia, Lachnospirales order, Rikenellaceae family,* and *Rothia*, among others. Conversely, certain genera such as *Clostridium_T* and *Agathobaculum* appeared reduced in IgAN. Encouragingly, when mapping these genera to the species-level signals observed in our MR analysis, five genus-level signals (eg, *Alistipes_A_871400, Bacteroides_H, Clostridium_T, Ruminococcus_C_58660*, and *Rothia*) corresponded to species implicated in the MR study. These congruent findings provide additional empirical support for the derived genetic associations (Figure 4(f)).

Functional prediction analysis was conducted using PICRUSt2. Alpha diversity, as measured by Chao1 and Shannon indices, showed no significant differences between IgAN patients and healthy controls (Chao1 *p* = .75; Shannon *p* = .06) (Figure S2(a,b)). Similarly, beta diversity analysis using PCoA based on Bray–Curtis dissimilarity did not reveal a significant separation between groups (PERMANOVA *p* = .19; R^2^ = 0.01) (Figure S2(c)). PICRUSt2-based functional inference identified a number of microbial pathways that were differentially enriched between groups. Specifically, 29 pathways were significantly upregulated in IgAN patients, including those related to carbohydrate degradation (eg, D-glucarate, ketogluconate, and D-galactarate metabolism), fatty acid β-oxidation, amino acid and sulfur metabolism, and biosynthesis of cofactors and vitamins such as folate, biotin, and heme. In contrast, the urea cycle pathway was significantly downregulated in IgAN, suggesting altered microbial nitrogen metabolism (Figure S2(d), Table S7).

## 4. Discussion

The present study provides a comprehensive examination of the potential causal relationships between the gut microbiota and IgAN, generating valuable insights into how specific bacterial taxa may influence disease risk. By integrating MR analyses and publicly available 16S rRNA sequencing data, it was possible to combine genetic evidence with population-level microbiome profiles to address the long-standing question of whether the gut microbiota simply reflects changes in IgAN or actively contributes to disease susceptibility. From the initial two-sample MR analysis using IVW method, nine gut microbial species emerged as significant, some showing a potential protective role – *s_Alistipes_senegalensis, s_Ruminococcus_bromii, and s_Bilophila_unclassified* – while others, including *s_Bacteroides_clarus*, appeared to elevate the risk of IgAN. Sensitivity analyses found little evidence of pleiotropy or heterogeneity, reinforcing the stability of these genetic associations. Furthermore, no reverse causation was detected, indicating that the uncovered relationships are unlikely to arise from the disease itself altering gut microbial composition. The findings regarding *s_Alistipes_senegalensis, s_Bacteroides_clarus,* and *s_Bilophila_unclassified* were especially robust when analyzed through multivariable MR, underscoring their independent contributions even when other gut microbes were jointly considered. This lends greater depth to the perspective that IgAN susceptibility may hinge on a fine interplay among multiple bacterial species, rather than a single dominant microbe.

Interestingly, our MR analysis suggested that *s_Alistipes senegalensis* has a protective effect against IgAN, yet gut microbiota profiling showed that *Alistipes* abundance was increased in patients. This apparent inconsistency can be attributed to several factors. First, the two analyses capture different aspects of microbial contribution to disease: MR estimates the effect of genetically predicted microbial abundance over a lifetime, which is less susceptible to reverse causality, while 16S rRNA sequencing provides a cross-sectional snapshot that may be influenced by disease progression, treatment, or host immune responses. Second, and more importantly, the validation data were derived at the genus level, whereas the MR analysis was performed at the species level. The genus *Alistipes* comprises multiple species that may have divergent or even opposing functional roles—some potentially protective, others pro-inflammatory. Therefore, the observed increase in genus-level *Alistipes* abundance in patients does not necessarily contradict the species-level protective association of A. senegalensis. Rather, this highlights the biological heterogeneity within the genus, and suggests that *Alistipes* as a genus plays a functionally relevant and non-neutral role in the pathophysiology of IgAN.The protective effects of *s_Alistipes_senegalensis* resonate with broader observations that certain *Alistipes* strains can modulate numerous physiological processes, including intestinal inflammation, lipid metabolism, and the gut epithelial barrier, in ways potentially beneficial to systemic homeostasis [25]. Alterations in the *Alistipes* genus have been reported in various diseases, sometimes exerting beneficial roles by regulating host urate excretion or controlling local immune responses to limit chronic inflammatory cascades [26,27]. *Alistipes_A_871400* showed significant enrichment at the genus level among IgAN patients, corroborating previous findings [28]. Interestingly, members of the *Alistipes* genus can exert both beneficial and adverse effects: for example, *Alistipes indistinctus* may mitigate hyperuricemia through hippuric acid production [29], whereas *Alistipes inops* has been implicated in disrupted tryptophan metabolism and exacerbated depressive symptoms [30]. In the context of IgAN, such protective actions could stem from the ability to fortify mucosal immunity, thereby reducing the formation or deposition of nephrotoxic immune complexes [27]. Indeed, IgAN pathogenesis is believed to involve complex IgA-type immune complexes that deposit in the glomerular mesangium, often triggered by defective mucosal immunity in the gut or respiratory tracts [31,32]. It is plausible that commensal strains like *s_Alistipes_senegalensis* might strengthen gut-barrier integrity or shape immune tolerance, mitigating the entry or formation of pathogenic IgA complexes. Additional mechanistic studies (eg, metagenomic, metatranscriptomic, or metabolomic assessments) would be instrumental in delineating precisely how these bacteria act at a molecular level [33], potentially revealing therapeutic applications such as dietary interventions or probiotic supplementation tailored to preserve beneficial *Alistipes* strains [34].

Conversely, *s_Bacteroides_clarus* was linked to higher IgAN susceptibility, which aligns with the recognized role of *Bacteroides* species in modulating local IgA production and intestinal immunity [35]. The genus *Bacteroides* is known for its immense diversity, with some strains fostering epithelial barrier integrity [36], while others provoke pro-inflammatory signaling [37]. The current analysis suggests that at least one *Bacteroides* species might engage in immunological pathways that exacerbate IgAN development. In IgA-deficient mice, *Bacteroides uniformis* becomes disrupted in its native gut habitat, ultimately exacerbating inflammation within the intestinal tract [38]. Mechanistically, *Bacteroides* taxa can stimulate T cell-dependent immunoglobulin class switching, possibly leading to altered IgA glycosylation patterns in genetically predisposed individuals [39]. In IgAN, aberrantly glycosylated IgA1 is central to pathogenic immune complex formation, and gut microbes have been implicated in driving this process through cross-reactive mucosal responses. Future functional validations may focus on whether *s_Bacteroides_clarus* produces specific metabolites or elicits certain T helper cell subsets that amplify mesangial deposition of IgA. Clarifying these pathways would open avenues for new preventive or therapeutic strategies, for instance selective targeting of harmful *Bacteroides* subtypes using bacteriophage therapies, antibiotics with narrow spectra, or engineered probiotics capable of competitive exclusion.

By integrating publicly available 16S rRNA data from multiple cohorts and applying the ConQuR approach to remove batch effects, this study provides further real-world support for the identified associations, showing that the genus-level taxa corresponding to five of the nine MR-derived species were indeed altered in IgAN patients. This convergence of genetic, epidemiologic, and compositional evidence strengthens the case that the identified species are not spurious findings attributable to confounding variables, geographic differences, or platform-specific biases. Instead, the results point to consistent, biologically plausible patterns of microbial disruptions in IgAN. While alpha diversity did not vary appreciably between IgAN and healthy controls, beta diversity metrics, such as Bray-Curtis dissimilarity, indicated significant compositional changes, underscoring the notion that shifts in particular microbes—and not necessarily overall richness—may be integral to disease manifestation [40]. This fits within the more nuanced view of gut dysbiosis, in which qualitative changes in specific microbial populations can drive pathological immune or metabolic outcomes.

The present findings also compare favorably with earlier MR efforts that investigated IgAN or related autoimmune conditions but primarily used broad-level taxonomic classifications [41–43]. Previous analyses have generally focused on the genus or phylum level, sometimes overlooking the fact that closely related species might have diametrically opposite effects. By identifying associations at the species level, this study increases precision, highlighting targets more suitable for rational intervention. Moreover, the inclusion of MVMR extends beyond single-exposure analyses, accounting for the interplay of multiple microbial exposures that typically coexist in the gut microbiome. Such a design enhances causal specificity, revealing which species exert truly independent effects after controlling for correlated species. To further contextualize the differences between our results and previous studies, we provide a side-by-side comparison of study designs and data sources in Table S6. Differences in GWAS sample sizes, ancestry, and microbiome reference databases likely contributed to the discrepancies in identified taxa. These findings provide a more refined and methodologically robust understanding of the gut–kidney axis, advancing from broad observational associations toward specific, mechanistically testable hypotheses.

A distinctive aspect of this work is the mediation analysis focusing on serum IgA levels, a central player in IgAN pathogenesis. Although none of the identified microbes displayed a direct influence on serum IgA levels, this outcome should not negate their possible involvement in IgA dysfunction. The pathophysiology of IgAN is intertwined with gut mucosal immunity, altered galactosylation of IgA1, and dysregulated IgA production [44]. The concluding result of no direct causal pathway *via* serum IgA suggests that the effects of *s_Alistipes_senegalensis, s_Bacteroides_clarus*, or other taxa on IgAN risk may occur through alternative immunological or metabolic routes rather than simply upregulating or downregulating IgA in circulation. It is conceivable, for example, that local IgA2 responses in the gut or the glycosylation state of secretory IgA could play a more prominent role than total serum IgA concentration *per se*. Further investigations that assess IgA subclass distribution, glycosylation profiles, and the role of microbial metabolites in local mucosal compartments might clarify these more intricate pathways.

Additionally, these results could inform clinical practice and future research in numerous ways. First, screening for detrimental microbes such as *s_Bacteroides_clarus* or beneficial strains like *s_Alistipes_senegalensis* might facilitate early risk assessment and prophylactic measures in at-risk populations. Second, interventions that selectively modulate gut microbiota—be it through precision probiotics [45], fecal microbiota transplantation [46], or targeted antimicrobials [47]—could hold promise for mitigating disease progression. Unlike therapies that broadly suppress the immune system, microbiota-focused interventions might preserve beneficial immune functions while specifically neutralizing negative microbial triggers. Finally, follow-up prospective trials will need to confirm whether altering the abundance of these putative risk or protective species translates into clinically meaningful improvements in renal outcomes. Indeed, many processes in IgAN, such as mesangial proliferation or complement activation, may only partially be shaped by gut-derived antigens, so better understanding the synergy between microbial factors and host immunogenetics remains vital.

The observed enrichment of microbial metabolic pathways in IgAN patients suggests a shift toward a more metabolically active and immunologically reactive gut microbiota. The upregulation of pathways related to carbohydrate degradation, fatty acid β-oxidation, and methylglyoxal detoxification indicates increased microbial energy turnover and stress response. These changes may lead to altered production of metabolites such as short-chain fatty acids, aldehydes, and ketones, which are known to influence mucosal immune tone, intestinal permeability, and systemic inflammation [48]. Additionally, the enhanced potential for sulfur metabolism, cysteine biosynthesis, and cofactor/vitamin pathways (eg, biotin, folate, heme) suggests increased microbial engagement in redox regulation and host micronutrient modulation, both of which can affect IgA production and immune cell activation [49]. Of particular interest is the enrichment of the polymyxin resistance pathway, which may indicate an ecological shift favoring resilient or pro-inflammatory taxa, potentially disrupting immune tolerance and contributing to intestinal immune activation [50]. This aligns with previous findings linking dysbiosis-induced expansion of pathobionts to gut barrier dysfunction and systemic immune priming, both relevant to IgAN pathogenesis [51]. In contrast, the decrease in the urea cycle pathway points to a potential impairment in microbial nitrogen metabolism, particularly in ammonia detoxification and arginine utilization. As arginine is a critical substrate for both microbial and host nitric oxide production, its dysregulation may compromise epithelial integrity, suppress regulatory T cell function, or enhance pro-inflammatory macrophage activity, all of which may exacerbate renal inflammation [52,53]. Moreover, impaired urea cycle activity could result in local or systemic accumulation of nitrogenous waste, which may further aggravate renal burden [54]. Together, these functional changes suggest that in IgAN, the gut microbiota is not only structurally altered.

Alternative biobank resources such as the UK Biobank, FinnGen, and the Japan Biobank (BJJ) were not utilized in this study due to several limitations that could compromise the reliability of findings for IgAN. The UK Biobank, while extensive, suffers from healthy volunteer bias and limited representation of non-European populations, which reduces its applicability to diseases like IgAN that exhibit significant ethnic variability in prevalence and genetic architecture. Similarly, FinnGen’s focus on the Finnish population introduces challenges related to founder effects and population-specific variants, limiting generalizability. Both biobanks also lack detailed clinical phenotyping or standardized diagnostic criteria for IgAN, increasing the risk of phenotype misclassification. Although the Japan Biobank provides large-scale genomic data from East Asian populations, its relatively small number of IgAN cases and insufficient phenotypic validation restrict statistical power and reliability. Importantly, previously identified GWAS loci for IgAN have not demonstrated significant replication within these datasets, raising concerns about their utility for uncovering novel pathogenic mechanisms. By selecting specialized datasets with stringent IgAN diagnostic criteria and sufficient case numbers, this study aimed to enhance both the accuracy and clinical relevance of its findings, ensuring a robust foundation for exploring causal mechanisms and identifying actionable therapeutic targets for IgAN.

Compared with previous MR studies on IgAN and related renal traits, our study offers a key advantage by integrating species-level causal inference with external validation across independent cohorts. Prior work was often limited to genus-level resolution, small sample sizes, or lacked replication. By combining genetic associations with real-world microbiota profiles and applying the ConQuR algorithm—a quantile regression-based method for controlling confounders and batch effects—we improved the comparability and robustness of microbial abundance estimates across heterogeneous datasets. Although we used publicly available microbiome datasets, we carefully selected those with stringent IgAN diagnostic criteria and adequate sample sizes to enhance reliability in real-world contexts. The concordance between MR-identified species and observational shifts at the genus level supports the biological plausibility of our findings. Several limitations should be acknowledged. First, GWAS-based instruments capture only a portion of microbiome heritability and do not directly reflect functional activity. Second, differences in ancestry between European GWAS (exposure) and East Asian microbiome cohorts (validation) may introduce heterogeneity, though consistent findings across populations suggest broader applicability. Third, the observed associations did not survive correction for multiple testing, which limits the strength of causal inference. Finally, lack of metadata on diet, medications, or comorbidities limited confounder adjustment in validation analyses. Nonetheless, the triangulation of MR, microbiome profiling, and PICRUSt2-based functional inference provides a multi-layered and internally consistent picture. Future research should build upon these strengths by incorporating multi-omics data, ancestry-matched cohorts, and deep phenotyping to elucidate functional mechanisms and support microbiota-targeted diagnostics or therapeutics for IgAN.

## 5. Conclusion

This study highlights a potential causal role of specific gut microbes in IgAN, identifying several taxa as protective or risk factors. Beyond taxonomic shifts, PICRUSt2-based functional prediction revealed metabolic alterations in IgAN, including enhanced carbohydrate and fatty acid metabolism and suppressed urea cycle activity, suggesting disruptions in nitrogen and energy processing. These findings emphasize the modifiable nature of the gut microbiome and its potential as a target for dietary, probiotic, or pharmacological interventions. The identified microbes and pathways may serve as biomarkers for risk stratification or therapeutic targets. As IgAN remains globally prevalent and clinically heterogeneous, integrating microbiome-based precision medicine could refine diagnosis and treatment. Although further mechanistic and interventional studies are needed, this work advances our understanding of gut–kidney crosstalk and supports the development of microbe-centered strategies for IgAN management.

## Supplementary Material

Supplementary- Clean.docx
