# Supplementary material for: Causal relationships between gut microbiota and IgA nephropathy: evidence from Mendelian randomization and microbiome validation
Source: Ren Fail. 2025 Jun 25;47(1):2522979. doi: 10.1080/0886022X.2025.2522979 (PMC12893488; doi:10.1080/0886022X.2025.2522979)
Supplement: Supplementary- Clean.docx [file IRNF_A_2522979_SM7338.docx]

**SUPPLEMENTARY MATERIAL**

Supplementary Tables

**Table S1** Instrument variants used in MR analysis

**Table S2** Mendelian randomization and sensitive analysis of the significant gut microbiota and IgA nephropathy

**Table S3** Results of the reverse Mendelian randomization between significant gut microbiota and IgA nephropathy

**Table S4** Mediation Mendelian randomization analysis result of the serum IgA between significant gut microbiota and IgA nephropathy

**Table S5** STROBE-MR checklist of recommended items to address in reports of Mendelian randomization studies

**Table S6** Comparison of Current and Previous Mendelian Randomization Studies on the Association Between Gut Microbiota and IgA Nephropathy

**Table S7** Mapping of Microbial Pathway IDs to Functional Descriptions

Supplementary Figure

**Figure S1** Causal effect estimates of gut microbiota on IgA nephropathy based on scatter plots

**Figure S2** Functional prediction of gut microbiota using PICRUSt2 in IgA nephropathy patients versus healthy controls

**Supplementary Table 1 Instrument variants used in MR analysis**

|  | **SNP** | **Effect_allele** | **Other_allele** | **Beta** | **Se** | **EAF** | **Chr** | **Pos** | **Pval** | **Samplesize** |
| --- | --- | --- | --- | --- | --- | --- | --- | --- | --- | --- |
| **g_Barnesiella** | \| rs10026787 \| \| --- \| \| rs1078988 \| \| rs115004061 \| \| rs118183316 \| \| rs11851037 \| \| rs34437635 \| \| rs62135856 \| \| rs67396441 \| \| rs6920861 \| \| rs700801 \| \| rs74448867 \| \| rs78335793 \| \| rs8029854 \| \| rs9884588 \| | \| C \| \| --- \| \| C \| \| T \| \| A \| \| T \| \| T \| \| A \| \| T \| \| C \| \| A \| \| G \| \| T \| \| C \| \| T \| | \| T \| \| --- \| \| T \| \| C \| \| G \| \| C \| \| C \| \| G \| \| C \| \| T \| \| G \| \| A \| \| C \| \| T \| \| C \| | \| -0.158 \| \| --- \| \| 0.094 \| \| -0.190 \| \| 0.177 \| \| 0.081 \| \| 0.162 \| \| 0.154 \| \| -0.079 \| \| -0.110 \| \| 0.101 \| \| 0.169 \| \| 0.136 \| \| -0.108 \| \| 0.083 \| | \| 0.034 \| \| --- \| \| 0.021 \| \| 0.043 \| \| 0.040 \| \| 0.018 \| \| 0.035 \| \| 0.033 \| \| 0.017 \| \| 0.021 \| \| 0.022 \| \| 0.035 \| \| 0.030 \| \| 0.022 \| \| 0.018 \| | \| 0.070 \| \| --- \| \| 0.258 \| \| 0.051 \| \| 0.052 \| \| 0.318 \| \| 0.072 \| \| 0.078 \| \| 0.386 \| \| 0.254 \| \| 0.813 \| \| 0.069 \| \| 0.095 \| \| 0.811 \| \| 0.371 \| | \| 4 \| \| --- \| \| 15 \| \| 3 \| \| 12 \| \| 14 \| \| 22 \| \| 2 \| \| 2 \| \| 6 \| \| 9 \| \| 8 \| \| 1 \| \| 15 \| \| 4 \| | \| 105512657 \| \| --- \| \| 101362495 \| \| 109716445 \| \| 30880725 \| \| 104301366 \| \| 44860850 \| \| 37836491 \| \| 127014797 \| \| 167527790 \| \| 76619731 \| \| 3946563 \| \| 175446149 \| \| 77021690 \| \| 32615152 \| | \| 3.395E-06 \| \| --- \| \| 4.865E-06 \| \| 8.764E-06 \| \| 9.721E-06 \| \| 9.555E-06 \| \| 3.907E-06 \| \| 4.118E-06 \| \| 5.049E-06 \| \| 1.550E-07 \| \| 5.802E-06 \| \| 1.280E-06 \| \| 4.490E-06 \| \| 1.670E-06 \| \| 3.938E-06 \| | \| 7738 \| \| --- \| \| 7738 \| \| 7738 \| \| 7738 \| \| 7738 \| \| 7738 \| \| 7738 \| \| 7738 \| \| 7738 \| \| 7738 \| \| 7738 \| \| 7738 \| \| 7738 \| \| 7738 \| |
| **s_Rothia_mucilaginosa** | \| rs17049177 \| \| --- \| \| rs616801 \| | \| A \| \| --- \| \| A \| | \| G \| \| --- \| \| G \| | \| -0.266 \| \| --- \| \| 0.240 \| | \| 0.054 \| \| --- \| \| 0.053 \| | \| 0.113 \| \| --- \| \| 0.120 \| | \| 2 \| \| --- \| \| 1 \| | \| 129157894 \| \| --- \| \| 94546142 \| | \| 8.394E-07 \| \| --- \| \| 6.911E-06 \| | \| 7738 \| \| --- \| \| 7738 \| |
| **s_Barnesiella_intestinihominis** | \| rs10026787 \| \| --- \| \| rs1078988 \| \| rs115004061 \| \| rs2132254 \| \| rs34437635 \| \| rs62135856 \| \| rs67396441 \| \| rs6920861 \| \| rs700801 \| \| rs74448867 \| \| rs78335793 \| \| rs8029854 \| \| rs9884588 \| | \| C \| \| --- \| \| C \| \| T \| \| T \| \| T \| \| A \| \| T \| \| C \| \| A \| \| G \| \| T \| \| C \| \| T \| | \| T \| \| --- \| \| T \| \| C \| \| C \| \| C \| \| G \| \| C \| \| T \| \| G \| \| A \| \| C \| \| T \| \| C \| | \| -0.157 \| \| --- \| \| 0.094 \| \| -0.190 \| \| 0.081 \| \| 0.162 \| \| 0.153 \| \| -0.079 \| \| -0.111 \| \| 0.101 \| \| 0.168 \| \| 0.135 \| \| -0.109 \| \| 0.083 \| | \| 0.034 \| \| --- \| \| 0.021 \| \| 0.043 \| \| 0.018 \| \| 0.035 \| \| 0.033 \| \| 0.017 \| \| 0.021 \| \| 0.022 \| \| 0.035 \| \| 0.030 \| \| 0.022 \| \| 0.018 \| | \| 0.070 \| \| --- \| \| 0.258 \| \| 0.051 \| \| 0.589 \| \| 0.072 \| \| 0.078 \| \| 0.386 \| \| 0.254 \| \| 0.813 \| \| 0.069 \| \| 0.095 \| \| 0.811 \| \| 0.371 \| | \| 4 \| \| --- \| \| 15 \| \| 3 \| \| 3 \| \| 22 \| \| 2 \| \| 2 \| \| 6 \| \| 9 \| \| 8 \| \| 1 \| \| 15 \| \| 4 \| | \| 105512657 \| \| --- \| \| 101362495 \| \| 109716445 \| \| 65861184 \| \| 44860850 \| \| 37836491 \| \| 127014797 \| \| 167527790 \| \| 76619731 \| \| 3946563 \| \| 175446149 \| \| 77021690 \| \| 32615152 \| | \| 3.933E-06 \| \| --- \| \| 5.589E-06 \| \| 8.833E-06 \| \| 9.738E-06 \| \| 3.749E-06 \| \| 4.396E-06 \| \| 5.506E-06 \| \| 1.049E-07 \| \| 5.742E-06 \| \| 1.429E-06 \| \| 5.674E-06 \| \| 1.212E-06 \| \| 3.897E-06 \| | \| 7738 \| \| --- \| \| 7738 \| \| 7738 \| \| 7738 \| \| 7738 \| \| 7738 \| \| 7738 \| \| 7738 \| \| 7738 \| \| 7738 \| \| 7738 \| \| 7738 \| \| 7738 \| |
| **s_Alistipes_senegalensis** | \| rs13381162 \| \| --- \| \| rs13393033 \| \| rs4935713 \| \| rs56168514 \| \| rs592284 \| \| rs7155195 \| \| rs73083250 \| \| rs7944200 \| \| rs852540 \| \| rs9573198 \| | \| T \| \| --- \| \| T \| \| T \| \| G \| \| T \| \| C \| \| A \| \| T \| \| A \| \| C \| | \| G \| \| --- \| \| C \| \| C \| \| A \| \| G \| \| T \| \| G \| \| C \| \| G \| \| G \| | \| 0.090 \| \| --- \| \| -0.088 \| \| 0.094 \| \| 0.141 \| \| -0.125 \| \| -0.091 \| \| -0.100 \| \| -0.086 \| \| 0.138 \| \| 0.104 \| | \| 0.020 \| \| --- \| \| 0.019 \| \| 0.021 \| \| 0.030 \| \| 0.028 \| \| 0.019 \| \| 0.022 \| \| 0.018 \| \| 0.031 \| \| 0.023 \| | \| 0.320 \| \| --- \| \| 0.338 \| \| 0.234 \| \| 0.098 \| \| 0.882 \| \| 0.572 \| \| 0.208 \| \| 0.596 \| \| 0.089 \| \| 0.181 \| | \| 18 \| \| --- \| \| 2 \| \| 10 \| \| 12 \| \| 10 \| \| 14 \| \| 3 \| \| 11 \| \| 7 \| \| 13 \| | \| 59277810 \| \| --- \| \| 6578442 \| \| 50499982 \| \| 89528844 \| \| 115372538 \| \| 85580506 \| \| 196224520 \| \| 28680730 \| \| 5552805 \| \| 73434771 \| | \| 3.665E-06 \| \| --- \| \| 3.075E-06 \| \| 8.015E-06 \| \| 2.129E-06 \| \| 8.746E-06 \| \| 9.582E-07 \| \| 7.028E-06 \| \| 1.368E-06 \| \| 9.709E-06 \| \| 6.818E-06 \| | \| 7738 \| \| --- \| \| 7738 \| \| 7738 \| \| 7738 \| \| 7738 \| \| 7738 \| \| 7738 \| \| 7738 \| \| 7738 \| \| 7738 \| |
| **s_Clostridium_asparagiforme** | \| rs11060559 \| \| --- \| \| rs12441278 \| \| rs12633258 \| \| rs13166845 \| \| rs1463878 \| \| rs1976629 \| \| rs2063471 \| \| rs35595126 \| \| rs35665752 \| \| rs57193778 \| \| rs574764 \| \| rs6597377 \| \| rs715519 \| \| rs73187354 \| \| rs80196726 \| | \| A \| \| --- \| \| A \| \| A \| \| A \| \| G \| \| T \| \| C \| \| T \| \| G \| \| G \| \| A \| \| C \| \| C \| \| A \| \| G \| | \| G \| \| --- \| \| T \| \| G \| \| T \| \| T \| \| C \| \| G \| \| C \| \| T \| \| A \| \| C \| \| A \| \| G \| \| G \| \| C \| | \| 0.198 \| \| --- \| \| 0.193 \| \| -0.368 \| \| 0.281 \| \| 0.208 \| \| -0.303 \| \| 0.182 \| \| 0.284 \| \| -0.283 \| \| 0.274 \| \| 0.189 \| \| -0.183 \| \| -0.259 \| \| -0.243 \| \| -0.295 \| | \| 0.044 \| \| --- \| \| 0.040 \| \| 0.081 \| \| 0.062 \| \| 0.045 \| \| 0.067 \| \| 0.039 \| \| 0.063 \| \| 0.062 \| \| 0.061 \| \| 0.041 \| \| 0.040 \| \| 0.057 \| \| 0.050 \| \| 0.066 \| | \| 0.742 \| \| --- \| \| 0.428 \| \| 0.069 \| \| 0.138 \| \| 0.780 \| \| 0.906 \| \| 0.455 \| \| 0.114 \| \| 0.107 \| \| 0.110 \| \| 0.386 \| \| 0.453 \| \| 0.140 \| \| 0.184 \| \| 0.093 \| | \| 12 \| \| --- \| \| 15 \| \| 3 \| \| 5 \| \| 3 \| \| 18 \| \| 4 \| \| 14 \| \| 1 \| \| 8 \| \| 18 \| \| 6 \| \| 22 \| \| 12 \| \| 4 \| | \| 129799240 \| \| --- \| \| 99523430 \| \| 60678910 \| \| 165663255 \| \| 192895132 \| \| 78718271 \| \| 59462687 \| \| 64759326 \| \| 28421400 \| \| 135705844 \| \| 63974140 \| \| 8991653 \| \| 50599940 \| \| 107648020 \| \| 19448339 \| | \| 8.008E-06 \| \| --- \| \| 1.345E-06 \| \| 5.133E-06 \| \| 6.550E-06 \| \| 4.375E-06 \| \| 5.600E-06 \| \| 3.446E-06 \| \| 7.319E-06 \| \| 5.210E-06 \| \| 7.017E-06 \| \| 3.777E-06 \| \| 4.583E-06 \| \| 5.576E-06 \| \| 1.397E-06 \| \| 8.676E-06 \| | \| 7738 \| \| --- \| \| 7738 \| \| 7738 \| \| 7738 \| \| 7738 \| \| 7738 \| \| 7738 \| \| 7738 \| \| 7738 \| \| 7738 \| \| 7738 \| \| 7738 \| \| 7738 \| \| 7738 \| \| 7738 \| |
| **s_Ruminococcus_bromii** | \| rs10864073 \| \| --- \| \| rs12041621 \| \| rs12131104 \| \| rs1884673 \| \| rs34762184 \| \| rs4884323 \| \| rs9865704 \| | \| C \| \| --- \| \| A \| \| T \| \| T \| \| A \| \| G \| \| A \| | \| T \| \| --- \| \| G \| \| C \| \| C \| \| G \| \| T \| \| G \| | \| -0.136 \| \| --- \| \| -0.128 \| \| 0.133 \| \| -0.150 \| \| -0.129 \| \| -0.092 \| \| -0.145 \| | \| 0.031 \| \| --- \| \| 0.026 \| \| 0.030 \| \| 0.030 \| \| 0.029 \| \| 0.020 \| \| 0.030 \| | \| 0.116 \| \| --- \| \| 0.199 \| \| 0.162 \| \| 0.123 \| \| 0.142 \| \| 0.398 \| \| 0.127 \| | \| 1 \| \| --- \| \| 1 \| \| 1 \| \| 20 \| \| 3 \| \| 13 \| \| 3 \| | \| 213838413 \| \| --- \| \| 48088907 \| \| 149917787 \| \| 39888893 \| \| 50504338 \| \| 59236358 \| \| 149362224 \| | \| 8.662E-06 \| \| --- \| \| 9.172E-07 \| \| 8.819E-06 \| \| 6.653E-07 \| \| 6.378E-06 \| \| 3.895E-06 \| \| 1.352E-06 \| | \| 7738 \| \| --- \| \| 7738 \| \| 7738 \| \| 7738 \| \| 7738 \| \| 7738 \| \| 7738 \| |
| **s_Bilophila_unclassified** | \| rs11067415 \| \| --- \| \| rs11260998 \| \| rs12913320 \| \| rs13009600 \| \| rs2579005 \| \| rs2990682 \| \| rs6127771 \| \| rs6958516 \| \| rs765025 \| \| rs77848425 \| | \| C \| \| --- \| \| A \| \| T \| \| A \| \| G \| \| G \| \| T \| \| C \| \| G \| \| T \| | \| A \| \| --- \| \| G \| \| C \| \| G \| \| T \| \| C \| \| C \| \| G \| \| C \| \| C \| | \| -0.095 \| \| --- \| \| -0.079 \| \| -0.093 \| \| 0.081 \| \| 0.091 \| \| 0.092 \| \| -0.080 \| \| -0.089 \| \| 0.103 \| \| 0.130 \| | \| 0.021 \| \| --- \| \| 0.018 \| \| 0.021 \| \| 0.017 \| \| 0.019 \| \| 0.019 \| \| 0.017 \| \| 0.019 \| \| 0.023 \| \| 0.028 \| | \| 0.221 \| \| --- \| \| 0.391 \| \| 0.237 \| \| 0.383 \| \| 0.701 \| \| 0.304 \| \| 0.535 \| \| 0.306 \| \| 0.167 \| \| 0.107 \| | \| 12 \| \| --- \| \| 1 \| \| 15 \| \| 2 \| \| 2 \| \| 1 \| \| 20 \| \| 7 \| \| 4 \| \| 4 \| | \| 115113185 \| \| --- \| \| 18391811 \| \| 88834557 \| \| 210907900 \| \| 215620045 \| \| 13512887 \| \| 38331330 \| \| 158447910 \| \| 65734627 \| \| 185636573 \| | \| 3.889E-06 \| \| --- \| \| 7.252E-06 \| \| 8.576E-06 \| \| 3.820E-06 \| \| 2.545E-06 \| \| 9.775E-07 \| \| 3.681E-06 \| \| 2.843E-06 \| \| 7.681E-06 \| \| 5.197E-06 \| | \| 7738 \| \| --- \| \| 7738 \| \| 7738 \| \| 7738 \| \| 7738 \| \| 7738 \| \| 7738 \| \| 7738 \| \| 7738 \| \| 7738 \| |
| **s_Bacteroides_clarus** | \| rs1157623 \| \| --- \| \| rs11676681 \| \| rs1470205 \| \| rs1667938 \| \| rs3756337 \| \| rs450741 \| \| rs4867972 \| \| rs814760 \| | \| A \| \| --- \| \| A \| \| T \| \| T \| \| C \| \| A \| \| G \| \| A \| | \| G \| \| --- \| \| G \| \| C \| \| C \| \| G \| \| C \| \| C \| \| G \| | \| 0.259 \| \| --- \| \| -0.164 \| \| -0.173 \| \| -0.261 \| \| -0.156 \| \| -0.346 \| \| -0.181 \| \| 0.188 \| | \| 0.059 \| \| --- \| \| 0.035 \| \| 0.034 \| \| 0.058 \| \| 0.035 \| \| 0.077 \| \| 0.038 \| \| 0.041 \| | \| 0.089 \| \| --- \| \| 0.590 \| \| 0.593 \| \| 0.094 \| \| 0.478 \| \| 0.939 \| \| 0.291 \| \| 0.216 \| | \| 3 \| \| --- \| \| 2 \| \| 3 \| \| 18 \| \| 5 \| \| 22 \| \| 5 \| \| 1 \| | \| 193034856 \| \| --- \| \| 33150881 \| \| 77267537 \| \| 77163994 \| \| 140806853 \| \| 21049682 \| \| 170505906 \| \| 110438037 \| | \| 9.948E-06 \| \| --- \| \| 2.144E-06 \| \| 5.062E-07 \| \| 6.798E-06 \| \| 6.485E-06 \| \| 7.119E-06 \| \| 1.540E-06 \| \| 5.670E-06 \| | \| 7738 \| \| --- \| \| 7738 \| \| 7738 \| \| 7738 \| \| 7738 \| \| 7738 \| \| 7738 \| \| 7738 \| |
| **s_Bacteroides_salyersiae** | \| rs117662076 \| \| --- \| \| rs12534702 \| \| rs1265300 \| \| rs12896430 \| \| rs17111728 \| \| rs3776996 \| \| rs76194327 \| \| rs9960494 \| | \| A \| \| --- \| \| T \| \| G \| \| A \| \| C \| \| A \| \| A \| \| C \| | \| G \| \| --- \| \| C \| \| A \| \| G \| \| T \| \| G \| \| T \| \| G \| | \| 0.404 \| \| --- \| \| 0.178 \| \| -0.178 \| \| -0.192 \| \| 0.334 \| \| 0.241 \| \| 0.380 \| \| -0.191 \| | \| 0.103 \| \| --- \| \| 0.022 \| \| -0.002 \| \| -0.026 \| \| 0.103 \| \| 0.040 \| \| -0.019 \| \| 0.003 \| | \| 0.055 \| \| --- \| \| 0.344 \| \| 0.503 \| \| 0.688 \| \| 0.071 \| \| 0.171 \| \| 0.064 \| \| 0.338 \| | \| 10 \| \| --- \| \| 7 \| \| 6 \| \| 14 \| \| 1 \| \| 5 \| \| 3 \| \| 18 \| | \| 119009986 \| \| --- \| \| 36729685 \| \| 4456166 \| \| 20963217 \| \| 55225711 \| \| 154417489 \| \| 116633349 \| \| 53680262 \| | \| 4.86048E-06 \| \| --- \| \| 8.54874E-06 \| \| 4.20388E-06 \| \| 4.79347E-06 \| \| 4.09959E-06 \| \| 1.16512E-06 \| \| 8.79296E-06 \| \| 6.76362E-06 \| | \| 7738 \| \| --- \| \| 7738 \| \| 7738 \| \| 7738 \| \| 7738 \| \| 7738 \| \| 7738 \| \| 7738 \| |

**Supplementary Table 2 Mendelian randomization and sensitive analysis of the significant gut microbiota and IgA nephropathy**

| **Exposure** | **Method** | **nSNP** | **beta** | **se** | **OR(95%CI)** | **P** | **Heterogeneity** |  | **Pleiotropy** |  | **FDR** |
| --- | --- | --- | --- | --- | --- | --- | --- | --- | --- | --- | --- |
|  |  |  |  |  |  |  | **Q statistic** | **Q_pval** | **Egger_intercept** | **Egger_pval** | **q-values** |
| g_Barnesiella | MR Egger | 14 | 0.973 | 0.469 | 2.65 (1.06-6.63) | 0.06 | 10.884 | 0.621 | -0.081 | 0.149 | 0.929 |
|  | Weighted median | 14 | 0.197 | 0.182 | 1.22 (0.85-1.74) | 0.28 |  |  |  |  |  |
|  | IVW  Simple mode  Weighted mode | 14  14  14 | 0.279  0.162  0.100 | 0.128  0.276  0.257 | 1.32 (1.03-1.70)  1.18 (0.68-2.02)  1.11 (0.67-1.83) | 0.03  0.57  0.70 |  |  |  |  |  |
| s_Rothia_mucilaginosa | IVW | 2 | 0.419 | 0.206 | 1.52 (1.02-2.28) | 0.04 | 1.280 | 0.258 | NA | NA | 0.929 |
| s_Barnesiella_intestinihominis | MR Egger | 13 | 0.990 | 0.477 | 2.69 (1.06-6.86) | 0.06 | 10.816 | 0.545 | -0.083 | 0.150 | 0.929 |
|  | Weighted median | 13 | 0.214 | 0.185 | 1.24 (0.86-1.78) | 0.25 |  |  |  |  |  |
|  | IVW  Simple mode  Weighted mode | 13  13  13 | 0.280  0.192  0.132 | 0.130  0.298  0.281 | 1.32 (1.03-1.71)  1.21 (0.68-2.17)  1.14 (0.66-1.98) | 0.03  0.53  0.65 |  |  |  |  |  |
| s_Alistipes_senegalensis | MR Egger | 10 | -0.079 | 1.045 | 0.92 (0.12-7.17) | 0.94 | 3.541 | 0.896 | -0.035 | 0.738 | 0.803 |
|  | Weighted median | 10 | -0.425 | 0.206 | 0.65 (0.44-0.98) | 0.04 |  |  |  |  |  |
|  | IVW  Simple mode  Weighted mode | 10  10  10 | -0.440  -0.406  -0.397 | 0.151  0.280  0.278 | 0.64 (0.48-0.87)  0.67 (0.39-1.15)  0.67 (0.39-1.16) | <0.01  0.18  0.19 |  |  |  |  |  |
| s_Clostridium_asparagiforme | MR Egger | 15 | 0.261 | 0.320 | 1.30 (0.69-2.43) | 0.44 | 5.319 | 0.806 | -0.021 | 0.792 | 0.803 |
|  | Weighted median | 15 | 0.214 | 0.092 | 1.24 (1.03-1.49) | 0.02 |  |  |  |  |  |
|  | IVW  Simple mode  Weighted mode | 15  15  15 | 0.175  0.215  0.232 | 0.068  0.152  0.141 | 1.19 (1.04-1.36)  1.24 (0.92-1.67)  1.26 (0.96-1.66) | 0.01  0.19  0.13 |  |  |  |  |  |
| s_Ruminococcus_bromii | MR Egger | 7 | -0.566 | 0.791 | 0.57 (0.12-2.68) | 0.51 | 5.205 | 0.518 | 0.035 | 0.741 | 0.929 |
|  | Weighted median | 7 | -0.280 | 0.196 | 0.76 (0.51-1.11) | 0.15 |  |  |  |  |  |
|  | IVW  Simple mode  Weighted mode | 7  7  7 | -0.293  -0.293  -0.297 | 0.139  0.304  0.288 | 0.75 (0.57-0.98)  0.75 (0.41-1.36)  0.74 (0.42-1.31) | 0.04  0.37  0.34 |  |  |  |  |  |
| s_Bilophila_unclassified | MR Egger | 10 | 1.019 | 1.178 | 2.77 (0.28-27.86) | 0.43 | 6.831 | 0.337 | -0.126 | 0.279 | 0.929 |
|  | Weighted median | 10 | -0.168 | 0.256 | 0.85 (0.51-1.39) | 0.51 |  |  |  |  |  |
|  | IVW  Simple mode  Weighted mode | 10  10  10 | -0.392  -0.082  -0.108 | 0.189  0.378  0.346 | 0.68 (0.47-0.98)  0.92 (0.44-1.93)  0.90 (0.46-1.77) | 0.04  0.84  0.77 |  |  |  |  |  |
| s_Bacteroides_salyersiae | MR Egger | 6 | 0.526 | 0.325 | 1.69 (0.89-3.20) | 0.18 | 1.641 | 0.896 | -0.081 | 0.310 | 0.929 |
|  | Weighted median | 6 | 0.141 | 0.100 | 1.15 (0.95-1.40) | 0.16 |  |  |  |  |  |
|  | IVW  Simple mode  Weighted mode | 6  6  6 | 0.159  0.147  0.143 | 0.078  0.128  0.129 | 1.17 (1.01-1.37)  1.16 (0.90-1.49)  1.15 (0.89-1.49) | 0.04  0.31  0.32 |  |  |  |  |  |
| s_Bacteroides_clarus | MR Egger | 8 | -0.011 | 0.399 | 0.99 (0.45-2.16) | 0.98 | 2.925 | 0.712 | 0.037 | 0.667 | 0.929 |
|  | Weighted median | 8 | 0.149 | 0.109 | 1.16 (0.94-1.44) | 0.17 |  |  |  |  |  |
|  | IVW  Simple mode  Weighted mode | 8  8  8 | 0.170  0.161  0.156 | 0.085  0.143  0.135 | 1.19 (1.00-1.40)  1.17 (0.89-1.55)  1.17 (0.90-1.52) | 0.05  0.31  0.30 |  |  |  |  |  |

**Supplementary Table 3 Results of the reverse Mendelian randomization between significant gut microbiota and IgA nephropathy**

| **Exposure** | **Outcome** | **Method** | **nSNP** | **beta** | **se** | **pval** | **OR** | **OR_lci95** | **OR_uci95** |
| --- | --- | --- | --- | --- | --- | --- | --- | --- | --- |
| IgAN | g_Barnesiella | MR Egger | 33 | -0.034405 | 0.041832 | 0.417097 | 0.96618 | 0.890123 | 1.048736 |
|  |  | Weighted median | 33 | 0.02055 | 0.02634 | 0.435278 | 1.020763 | 0.969401 | 1.074846 |
|  |  | Inverse variance weighted | 33 | 0.018957 | 0.0181 | 0.294948 | 1.019137 | 0.983616 | 1.055941 |
|  | s_Alistipes_senegalensis | MR Egger | 29 | -0.023925 | 0.044182 | 0.592599 | 0.976359 | 0.895367 | 1.064678 |
|  |  | Weighted median | 29 | -0.005107 | 0.02754 | 0.852893 | 0.994906 | 0.942626 | 1.050086 |
|  |  | Inverse variance weighted | 29 | -0.011436 | 0.019318 | 0.553844 | 0.988629 | 0.951896 | 1.026779 |
|  | s_Bacteroides_clarus | MR Egger | 33 | 0.044916 | 0.084084 | 0.597033 | 1.045939 | 0.887018 | 1.233334 |
|  |  | Weighted median | 33 | 0.056734 | 0.051556 | 0.271148 | 1.058374 | 0.956651 | 1.170913 |
|  |  | Inverse variance weighted | 33 | 0.010772 | 0.036104 | 0.765419 | 1.010831 | 0.941772 | 1.084953 |
|  | s_Bacteroides_salyersiae | MR Egger | 33 | 0.106873 | 0.099696 | 0.291998 | 1.112793 | 0.915275 | 1.352936 |
|  |  | Weighted median | 33 | 0.051146 | 0.057819 | 0.376381 | 1.052477 | 0.939714 | 1.17877 |
|  |  | Inverse variance weighted | 33 | 0.060696 | 0.042991 | 0.157994 | 1.062576 | 0.97671 | 1.155991 |
|  | s_Barnesiella_intestinihominis | MR Egger | 33 | -0.033441 | 0.041832 | 0.430131 | 0.967112 | 0.890982 | 1.049747 |
|  |  | Weighted median | 33 | 0.020231 | 0.026152 | 0.439174 | 1.020437 | 0.969449 | 1.074108 |
|  |  | Inverse variance weighted | 33 | 0.019424 | 0.0181 | 0.283202 | 1.019614 | 0.984076 | 1.056435 |
|  | s_Bilophila_unclassified | MR Egger | 33 | -0.016948 | 0.041099 | 0.682897 | 0.983194 | 0.9071 | 1.065672 |
|  |  | Weighted median | 33 | -0.013484 | 0.025373 | 0.595135 | 0.986607 | 0.938741 | 1.036913 |
|  |  | Inverse variance weighted | 33 | -0.010369 | 0.017824 | 0.560741 | 0.989685 | 0.955708 | 1.02487 |
|  | s_Clostridium_asparagiforme | MR Egger | 33 | 0.005024 | 0.115482 | 0.965579 | 1.005037 | 0.801459 | 1.260325 |
|  |  | Weighted median | 33 | 0.049054 | 0.060711 | 0.419095 | 1.050277 | 0.93245 | 1.182993 |
|  |  | Inverse variance weighted | 33 | 0.021813 | 0.049382 | 0.658691 | 1.022053 | 0.927766 | 1.125922 |
|  | s_Rothia_mucilaginosa | MR Egger | 33 | -0.026147 | 0.08411 | 0.757981 | 0.974192 | 0.82613 | 1.148789 |
|  |  | Weighted median | 33 | -0.053001 | 0.051735 | 0.305608 | 0.948379 | 0.856928 | 1.049589 |
|  |  | Inverse variance weighted | 33 | -0.03309 | 0.035855 | 0.356057 | 0.967451 | 0.901798 | 1.037885 |
|  | s_Ruminococcus_bromii | MR Egger | 32 | -0.065477 | 0.048141 | 0.18393 | 0.93662 | 0.852285 | 1.029301 |
|  |  | Weighted median | 32 | -0.007817 | 0.030459 | 0.797449 | 0.992213 | 0.934712 | 1.053252 |
|  |  | Inverse variance weighted | 32 | 0.003911 | 0.021102 | 0.852974 | 1.003918 | 0.963244 | 1.046311 |

**Supplementary Table 4 Mediation mendelian randomization analysis result of the serum IgA between significant gut microbiota and IgA nephropathy**

| **Exposure** | **gut_IgA level** | |  | **IgA level_IgAN** | | | **gut_IgAN** | | |  |
| --- | --- | --- | --- | --- | --- | --- | --- | --- | --- | --- |
|  | beta | se | p | beta | se | p | beta | se | p | |
| g_Barnesiella | 0.03 | 0.05 | 4.90E-01 | 0.89 | 0.28 | 1.78E-03 | 0.28 | 0.13 | 2.99E-02 | |
| g_Rothia | -0.01 | 0.05 | 8.40E-01 | 0.89 | 0.28 | 1.78E-03 | 0.42 | 0.21 | 4.19E-02 | |
| g_Barnesiella | 0.04 | 0.05 | 4.40E-01 | 0.89 | 0.28 | 1.78E-03 | 0.28 | 0.13 | 3.10E-02 | |
| g_Alistipes | 0.03 | 0.04 | 5.20E-01 | 0.89 | 0.28 | 1.78E-03 | -0.44 | 0.15 | 3.68E-03 | |
| g_Clostridium | 0.02 | 0.02 | 3.60E-01 | 0.89 | 0.28 | 1.78E-03 | 0.18 | 0.07 | 9.52E-03 | |
| g_Ruminococcus | -0.01 | 0.05 | 8.90E-01 | 0.89 | 0.28 | 1.78E-03 | -0.29 | 0.14 | 3.49E-02 | |
| g_Bilophila | -0.01 | 0.06 | 8.70E-01 | 0.89 | 0.28 | 1.78E-03 | -0.39 | 0.19 | 3.85E-02 | |
| s_Bacteroides_clarus | -0.01 | 0.03 | 8.00E-01 | 0.89 | 0.28 | 1.78E-03 | 0.17 | 0.08 | 4.45E-02 | |
| s_Bacteroides_salyersiae | 0.01 | 0.02 | 7.20E-01 | 0.89 | 0.28 | 1.78E-03 | 0.16 | 0.08 | 4.14E-02 | |

**Supplementary Table 5 STROBE-MR checklist of recommended items to address in reports of Mendelian randomization studies**^1^ ^2^

| **Item No.** | **Section** | **Checklist item** | **Page No.** | **Relevant text from manuscript** |
| --- | --- | --- | --- | --- |
| 1 | **TITLE and ABSTRACT** | Indicate Mendelian randomization (MR) as the study’s design in the title and/or the abstract if that is a main purpose of the study | 1-3 | Complete |
|  | **INTRODUCTION** |  |  |  |
| 2 | **Background** | Explain the scientific background and rationale for the reported study. What is the exposure? Is a potential causal relationship between exposure and outcome plausible? Justify why MR is a helpful method to address the study question | 4-5 | Complete –The relationship between gut microbiota and IgAN still unclear. We employ MR to explore their potential association in the introduction. |
| 3 | **Objectives** | State specific objectives clearly, including pre-specified causal hypotheses (if any). State that MR is a method that, under specific assumptions, intends to estimate causal effects | 4-5 | Complete – Objectives has been clarified in the paragraph 2 of the introduction. |
|  | **METHODS** |  |  |  |
| 4 | **Study design and data sources** | Present key elements of the study design early in the article. Consider including a table listing sources of data for all phases of the study. For each data source contributing to the analysis, describe the following: |  |  |
|  | a) | Setting: Describe the study design and the underlying population, if possible. Describe the setting, locations, and relevant dates, including periods of recruitment, exposure, follow-up, and data collection, when available. | 5 | Complete – All necessary information about the study design and underlying population has been described in the Material and methods section |
|  | b) | Participants: Give the eligibility criteria, and the sources and methods of selection of participants. Report the sample size, and whether any power or sample size calculations were carried out prior to the main analysis | 5 | Complete – All necessary information about GWAS studies been used in this study has been described in Material and methods section |
|  | c) | Describe measurement, quality control and selection of genetic variants | 5-6 | Complete – The genetic predictor selection process has been described in the Material and methods section “2.2. Statistical analysis and sensitivity analysis” |
|  | d) | For each exposure, outcome, and other relevant variables, describe methods of assessment and diagnostic criteria for diseases | 5 | Complete – The information about the diagnostic criteria for diseases has been described in the Material and methods section. |
|  | e) | Provide details of ethics committee approval and participant informed consent, if relevant | 17-18 | Complete – Ethical approval and participant informed consent has been described in the Ethics |
| 5 | **Assumptions** | Explicitly state the three core IV assumptions for the main analysis (relevance, independence and exclusion restriction) as well assumptions for any additional or sensitivity analysis | 5-6 | Complete – The Mendelian randomization assumptions have been described in the Material and methods section and Figure 1. |
| 6 | **Statistical methods: main analysis** | Describe statistical methods and statistics used |  |  |
|  | a) | Describe how quantitative variables were handled in the analyses (i.e., scale, units, model) | NA |  |
|  | b) | Describe how genetic variants were handled in the analyses and, if applicable, how their weights were selected | 5-6 | Complete – Described in the “2.1. GWAS database” and “2.2. Statistical analysis and sensitivity analysis” of the Material and methods section. |
|  | c) | Describe the MR estimator (e.g. two-stage least squares, Wald ratio) and related statistics. Detail the included covariates and, in case of two-sample MR, whether the same covariate set was used for adjustment in the two samples | 5-6 | Complete – Described in the “2.2. Statistical analysis and sensitivity analysis” of the Material and methods section. |
|  | d) | Explain how missing data were addressed | NA |  |
|  | e) | If applicable, indicate how multiple testing was addressed | NA |  |
| 7 | **Assessment of assumptions** | Describe any methods or prior knowledge used to assess the assumptions or justify their validity | 5-6 | Complete – Described in the “2.2. Statistical analysis and sensitivity analysis” of the Material and methods section. |
| 8 | **Sensitivity analyses and additional analyses** | Describe any sensitivity analyses or additional analyses performed (e.g. comparison of effect estimates from different approaches, independent replication, bias analytic techniques, validation of instruments, simulations) | 5-6 | Complete – Described in the “2.2. Statistical analysis and sensitivity analysis” and “2.3. Mediation analysis” of the Material and methods section. |
| 9 | **Software and pre-registration** |  |  |  |
|  | a) | Name statistical software and package(s), including version and settings used | 7-8 | Complete – The package and settings used are described in the Material and methods section. |
|  | b) | State whether the study protocol and details were pre-registered (as well as when and where) | NA |  |
|  | **RESULTS** |  |  |  |
| 10 | **Descriptive data** |  |  |  |
|  | a) | Report the numbers of individuals at each stage of included studies and reasons for exclusion. Consider use of a flow diagram |  | Complete – See Figure 1 |
|  | b) | Report summary statistics for phenotypic exposure(s), outcome(s), and other relevant variables (e.g. means, SDs, proportions) |  | Complete – See Figure 1 |
|  | c) | If the data sources include meta-analyses of previous studies, provide the assessments of heterogeneity across these studies | NA |  |
|  | d) | For two-sample MR:  i.  Provide justification of the similarity of the genetic variant-exposure associations between the exposure and outcome samples  ii.  Provide information on the number of individuals who overlap between the exposure and outcome studies | NA |  |
| 11 | **Main results** |  |  |  |
|  | a) | Report the associations between genetic variant and exposure, and between genetic variant and outcome, preferably on an interpretable scale |  | Complete –see Figure 2, Figure 4, Table S1,S2 and S3. |
|  | b) | Report MR estimates of the relationship between exposure and outcome, and the measures of uncertainty from the MR analysis, on an interpretable scale, such as odds ratio or relative risk per SD difference | NA |  |
|  | c) | If relevant, consider translating estimates of relative risk into absolute risk for a meaningful time period | NA |  |
|  | d) | Consider plots to visualize results (e.g. forest plot, scatterplot of associations between genetic variants and outcome versus between genetic variants and exposure) |  | Complete – See Figure 3. |
| 12 | **Assessment of assumptions** |  |  |  |
|  | a) | Report the assessment of the validity of the assumptions | 8-9 | Complete – We assessed the validity using IVW, MR-Egger, weighted median, simple mode, and weighted mode, as shown in the Results section |
|  | b) | Report any additional statistics (e.g., assessments of heterogeneity across genetic variants, such as *I^2^*, Q statistic or E-value) | 8 | Complete – We reported the use of Cochran’s Q in the Results section. |
| 13 | **Sensitivity analyses and additional analyses** |  |  |  |
|  | a) | Report any sensitivity analyses to assess the robustness of the main results to violations of the assumptions | 8-9 | Complete – We complemented IVW with MR-Egger and weighte as sensitivity analyses. See Figure 2 to 3. |
|  | b) | Report results from other sensitivity analyses or additional analyses | 8-10 | Complete – We conducted Cochran’s Q test.See Table S2 to S3 and Figure 2 to 4. |
|  | c) | Report any assessment of direction of causal relationship (e.g., bidirectional MR) | 8-10 | Complete –We performed two-way Mendelian randomization. Specific results are described in the results section. |
|  | d) | When relevant, report and compare with estimates from non-MR analyses | 9-10 | Complete – See Figure 5, Figure S1 and TableS7. |
|  | e) | Consider additional plots to visualize results (e.g., leave-one-out analyses) | NA |  |
|  | **DISCUSSION** |  |  |  |
| 14 | **Key results** | Summarize key results with reference to study objectives | 10-11 | Complete – Discussion paragraph 1. |
| 15 | **Limitations** | Discuss limitations of the study, taking into account the validity of the IV assumptions, other sources of potential bias, and imprecision. Discuss both direction and magnitude of any potential bias and any efforts to address them | 11-17 | Complete – Discussion. |
| 16 | **Interpretation** |  |  |  |
|  | a) | Meaning: Give a cautious overall interpretation of results in the context of their limitations and in comparison with other studies | 11-17 | Complete – Discussion. |
|  | b) | Mechanism: Discuss underlying biological mechanisms that could drive a potential causal relationship between the investigated exposure and the outcome, and whether the gene-environment equivalence assumption is reasonable. Use causal language carefully, clarifying that IV estimates may provide causal effects only under certain assumptions | 11-17 | Complete – Discussion. |
|  | c) | Clinical relevance: Discuss whether the results have clinical or public policy relevance, and to what extent they inform effect sizes of possible interventions | 11-17 | Complete – Discussion. |
| 17 | **Generalizability** | Discuss the generalizability of the study results (a) to other populations, (b) across other exposure periods/timings, and (c) across other levels of exposure | 11-17 | Complete – Discussion. |
|  | **OTHER INFORMATION** |  |  |  |
| 18 | **Funding** | Describe sources of funding and the role of funders in the present study and, if applicable, sources of funding for the databases and original study or studies on which the present study is based | 18 | Complete –See “Acknowledgements” section. |
| 19 | **Data and data sharing** | Provide the data used to perform all analyses or report where and how the data can be accessed, and reference these sources in the article. Provide the statistical code needed to reproduce the results in the article, or report whether the code is publicly accessible and if so, where | 18 | Complete – The data used in the study can be accessed and downloaded from original studies. |
| 20 | **Conflicts of Interest** | All authors should declare all potential conflicts of interest | 18 | Complete – All authors declare that they have no conflict of interest. |

This checklist is copyrighted by the Equator Network under the Creative Commons Attribution 3.0 Unported (CC BY 3.0) license.

1. Skrivankova VW, Richmond RC, Woolf BAR, Yarmolinsky J, Davies NM, Swanson SA, et al. Strengthening the Reporting of Observational Studies in Epidemiology using Mendelian Randomization (STROBE-MR) Statement. JAMA. 2021;under review.

2. Skrivankova VW, Richmond RC, Woolf BAR, Davies NM, Swanson SA, VanderWeele TJ, et al. Strengthening the Reporting of Observational Studies in Epidemiology using Mendelian Randomisation (STROBE-MR): Explanation and Elaboration. BMJ. 2021;375:n2233.

**Supplementary Table 6 Comparison of Current and Previous Mendelian Randomization Studies on the Association Between Gut Microbiota and IgA Nephropathy**

| Study | Summary data of gut microbiota | Summary data of IgA nephropathy | PMID | Time |
| --- | --- | --- | --- | --- |
| Previous studies 1 | MiBioGen consortium (211 bacterial taxa)  18340 European ancestry | FinnGen reseach (538 cases and 341,961 controls from Finnish population) | 37201114 | 2023 |
| Previous studies 2 | MiBioGen consortium (211 bacterial taxa) | A meta-analysis of 628,000 cross-population samples from the UK Biobank and FinnGen | 37242290 | 2023 |
| Current study | 7,738 participants (412 microbiotas) | 11 European cohorts (5,556 cases and 21,178 controls) |  |  |

**Supplementary Table 7 Mapping of Microbial Pathway IDs to Functional Descriptions.**

| ID | pathway |
| --- | --- |
| PWY0-41 | Folate transformations III |
| KETOGLUCONMET-PWY | Ketogluconate metabolism |
| GLUCARDEG-PWY | D-glucarate degradation I |
| PWY-5747 | 2-methylcitrate cycle II |
| PWY-7446 | Sulfoquinovose degradation I |
| PWY-5705 | Allantoin degradation to glyoxylate III |
| PWY0-42 | 2-methylcitrate cycle I |
| PWY0-1338 | Polymyxin resistance |
| METHGLYUT-PWY | Superpathway of methylglyoxal degradation |
| GALACTARDEG-PWY | D-galactarate degradation I |
| AST-PWY | L-arginine degradation II |
| GLUCARGALACTSUPER-PWY | Superpathway of D-glucarate and D-galactarate degradation |
| PWY0-1533 | Methylphosphonate degradation I |
| UBISYN-PWY | Superpathway of ubiquinol-8 biosynthesis |
| GLYCOL-GLYOXDEG-PWY | Superpathway of glycol metabolism and degradation |
| PWY-1861 | Formaldehyde assimilation II |
| RUMP-PWY | Formaldehyde oxidation I |
| FAO-PWY | Fatty acid β-oxidation I |
| PWY-5862 | Superpathway of demethylmenaquinol-9 biosynthesis |
| PWY0-1415 | Superpathway of heme b biosynthesis from uroporphyrinogen-III |
| SALVADEHYPOX-PWY | Adenosine nucleotides degradation II |
| PWY-5918 | Superpathay of heme biosynthesis from glutamate |
| SO4ASSIM-PWY | Assimilatory sulfate reduction I |
| FUC-RHAMCAT-PWY | Superpathway of fucose and rhamnose degradation |
| PWY-6608 | Guanosine nucleotides degradation III |
| PWY-6353 | Purine nucleotides degradation II |
| SULFATE-CYS-PWY | Superpathway of sulfate assimilation and cysteine biosynthesis |
| BIOTIN-BIOSYNTHESIS-PWY | Biotin biosynthesis I |
| PWY-6519 | 8-amino-7-oxononanoate biosynthesis I |
| PWY-4984 | Urea cycle |

**Figure S1 Causal effect estimates of gut microbiota on IgA nephropathy based on scatter plots.**

**Figure S1. Scatter plots for causal effects of gut microbiota on IgA nephropathy.** Abbreviations: MR, Mendelian Randomization; SNP, single-nucleotide polymorphism.

**Supplementary Figure S2 Functional prediction of gut microbiota using PICRUSt2 in IgA nephropathy patients versus healthy controls**

**Figure S2. Functional prediction of gut microbiota using PICRUSt2 from different batches between IgA nephropathy and healthy controls.** For a-b) alpha diversity was estimated using both the Chao1 index and the Shannon index. Two-sided Wilcoxon rank-sum test was used to assess the statistical significance. c) PERMANOVA analysis based on Bray-Curtis dissimilarity was performed to assess differences in beta diversity between groups. d) Differential pathway are shown (p < 0.05 and |log2FC| > log2(1.5); DESeq2).
